# Harnessing the Action Model of the Defense Responses Induced by UPSIDE^®^ Against *Plasmopara viticola* in Grapevine

**DOI:** 10.3390/plants15091297

**Published:** 2026-04-23

**Authors:** Giulia Scimone, Lorenzo D’Asaro, Zuzana Gelová, Lorenzo Cotrozzi, Lorenzo Mariotti, Lisa Milanollo, Claudia Pisuttu, Mariagrazia Tonelli, Elisa Pellegrini, Cristina Nali

**Affiliations:** 1 Department of Agriculture, Food and Environment, University of Pisa, Via del Borghetto 80, 56124 Pisa, Italy; giulia.scimone@phd.unipi.it (G.S.); lorenzo.dasaro@phd.unipi.it (L.D.); lorenzo.cotrozzi@unipi.it (L.C.); lorenzo.mariotti@unipi.it (L.M.); claudia.pisuttu@unipi.it (C.P.); mariagrazia.tonelli@unipi.it (M.T.); cristina.nali@unipi.it (C.N.); 2 Kwizda Agro, Universitätsring 6, 1010 Vienna, Austria; z.gelova@kwizda-agro.at (Z.G.); l.milanollo@kwizda-agro.at (L.M.)

**Keywords:** downy mildew, crop protection, *Saccharomyces cerevisiae*, priming, induced resistance, biotic stress

## Abstract

*Plasmopara viticola* (*Pv*), the causal agent of downy mildew, is one of the most damaging pathogens affecting grapevine. Current control strategies largely depend on copper-based fungicides and synthetic chemicals, raising increasing concerns related to environmental sustainability and pathogen resistance. This study evaluated the efficacy of a novel *Saccharomyces cerevisiae* extract (U) as an inducer of resistance in the grapevine–*Pv* interaction. Microscopic observations revealed the ability of U to inhibit *Pv* spread over the leaf. Additionally, biochemical and molecular responses were analyzed in grapevine leaves subjected to four treatments: plants treated only with water (U^−^/*Pv*^−^; i.e., control) or U (U^+^/*Pv*^−^), inoculated with *Pv* (U^−^/*Pv*^+^), or both treated with U and then inoculated with *Pv* (U^+^/*Pv*^+^). Fully expanded leaves were sampled at 2-, 5-, 24-, and 72-h post inoculation (hpi). In U^+^/*Pv*^−^ leaves, jasmonic, salicylic and abscisic acid (JA, SA, and ABA), as well as hydrogen peroxide (H_2_O_2_) increased at 2 hpi (+44, +33, +38%, and 3-fold, respectively), accompanied by upregulation of *pr1* (2-fold higher than control, respectively), suggesting the capacity of U to trigger the plant alert system. In U^−^/*Pv*^+^ leaves, peaks of JA and H_2_O_2_ occurred at 24 hpi (+40% and 4-fold higher than control), followed by marked ethylene emissions and upregulation of *pr1* and *pr2* (i.e., genes associated with *Pv* defense; around 2-fold, averagely) at 72 hpi, confirming the progression of infection. In contrast, U^+^/*Pv*^+^ leaves showed stronger peaks of H_2_O_2_ at both 2 and 5 hpi (7-fold and +58%, respectively), together with SA accumulation and upregulation of *pr1*, *pr2*, *eds1,* and *chit1b* at 72 hpi (more than 2-fold), suggesting a priming effect of U. Overall, U effectively enhanced grapevine defense responses and limited *Pv* development, highlighting its potential as a sustainable disease management strategy.

## 1. Introduction

Downy mildew caused by the oomycete *Plasmopara viticola* (*Pv*) represents one of the most destructive diseases affecting grapevines worldwide [1]. Given that *Vitis vinifera* L. is highly susceptible to *Pv* infection, effective management of this pathogen is essential to sustain grape and wine production [2]. Traditionally, the management of downy mildew has relied heavily on copper-based fungicides, particularly in organic viticulture, where the use of synthetic fungicides is prohibited. Copper, one of the oldest active ingredients employed in plant disease control, offers the advantage of a multi-site mode of action, which minimizes the risk of selecting fungicide-resistant strains [3]. However, copper applications may reach 15–17 treatments per season, especially in regions where climatic conditions favor the development of downy mildew. This intensive use leads to the progressive accumulation of copper in soil, as copper is a heavy metal that is neither biodegradable nor readily metabolized by soil microorganisms [4,5,6,7,8]. Moreover, long-term copper accumulation can cause significant microbiological and biochemical imbalances, including alterations in soil enzyme activity, suppression of beneficial microbial populations, and a decline in soil fertility [5]. Therefore, within vineyard ecosystems, the frequent use of copper-based fungicides represents a major environmental concern. In this context, it is also important to note that copper use in the European Union is subject to strict regulatory limits [maximum 28 kg/ha over a 7-year period, as established by Regulation (EU) 2018/1981], and, since 2025, copper compounds have been classified as “candidates for substitution”, further highlighting concerns about their long-term environmental impact. Beyond copper use in organic viticulture, conventional downy mildew management still largely relies on synthetic fungicides. However, their extensive and repeated application has been associated with adverse environmental impacts and the emergence of resistant pathogen strains [9,10]. In particular, the intensive use of site-specific fungicides can exert strong selective pressure on pathogen populations, leading to reduced efficacy over time and the need for increased application frequency or dosage. Furthermore, concerns have been raised regarding the persistence of these compounds in the environment, their potential effects on non-target organisms, and the contamination of soil and water resources. In response to these concerns, the European Union has introduced restrictions on chemical pesticide use, encouraging both research institutions and the agricultural industry to develop innovative and more sustainable strategies to address this ongoing challenge [11].

To promote environmentally sustainable viticulture, increasing attention has been directed toward promising alternatives derived from natural sources. In this context, the use of elicitors to stimulate plant immune responses represents a promising alternative to conventional fungicides. These molecules activate defense pathways in plants, leading to the production of antimicrobial compounds and/or reinforcement of the cell wall, thereby preventing or slowing pathogen invasion. Elicitors comprise a wide range of biochemical molecules (including lipids, proteins, and carbohydrates), many of which function as microbe-associated molecular patterns (MAMPs) and/or endogenous damage-associated molecular patterns (DAMPs) that are recognized by specialized receptors on plant cells [12]. Among them, laminarin, a β-glucan polysaccharide extracted from the brown alga *Laminaria digitata*, and its sulphated derivatives have demonstrated notable elicitor activity by stimulating plant defense responses [13,14]. Chitosan, another widely studied compound obtained through the alkaline deacetylation of chitin—a biopolymer found in the exoskeletons of crustaceans, insect cuticles, and fungal cell walls—has been shown to induce resistance against pathogens [15,16]. For instance, Mian et al. [17] reported that chitosan treatment can significantly reduce disease severity by upregulating genes involved in plant defense pathways, including those related to pathogenesis-related proteins and oxidative stress responses. Another biotechnological product, cerevisane, derived from purified cell walls of the yeast *Saccharomyces cerevisiae*, acts as an elicitor of systemic resistance pathways in plants [18]. Scimone et al. [18] demonstrated that yeast-derived products, specifically those obtained from *S. cerevisiae*, can induce a priming effect in plants, enhancing their readiness to respond to pathogen attack through activation of secondary metabolism, accumulation of defense-related compounds, and modulation of enzymatic activities. Overall, these findings support the role of cerevisane-based products as promising tools for sustainable disease management by strengthening the plant innate immune system rather than directly targeting the pathogen. This aspect is essential when optimizing application strategies for natural compounds and when designing new biopesticides derived from natural sources [19,20,21].

Therefore, the aim of this study was to evaluate the potential of a novel *S. cerevisiae* extract formulation named UPSIDE^®^, developed by Kwizda Agro GmbH (hereafter referred to as U; previously described in [18,22]), as an inducer of resistance in the *V. vinifera*-*Pv* pathosystem. Specifically, this study aimed to address the following questions: (i) Which hormonal mechanisms are activated in response to individual treatments (U^+^ or *Pv*^+^) and sequential double-treatment conditions (U^+^/*Pv*^+^)? (ii) Which defense-related genes may play a pivotal role in the grapevine adaptive response under single and sequential treatments? We postulate both a direct effect of U against *Pv* inoculation and an activation of systemic responses, including systemic acquired resistance (SAR; a long-lasting form of resistance against a broad spectrum of hemi- and/or biotrophic pathogens) and induced systemic resistance (ISR; an induced state of resistance triggered by both hemi-biotrophic and necrotrophic pathogens) [10,23,24] at the functional and metabolic levels.

## 2. Results

### 2.1. Effect of UPSIDE^®^ on Downy Mildew Severity by Leaf Disc Assay

Disease severity assessment showed that U reduced the development of downy mildew by 88% compared to water-sprayed leaves. Downy mildew affected, on average, 59% of the leaf disc surface, whereas in U-treated discs, disease severity was limited to 7% (Figure S1).

### 2.2. Microscopic Observations

**Figure 1:**
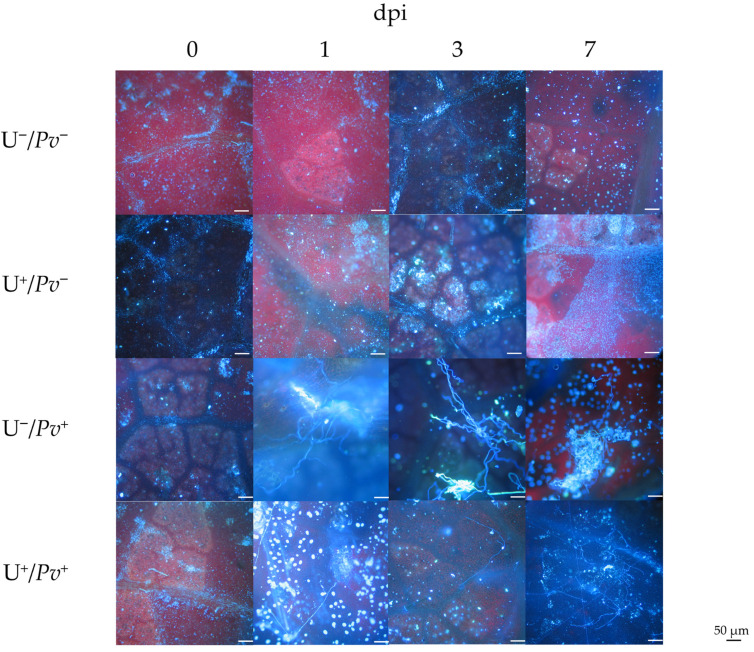
Leaves of *Vitis vinifera* cv. Sangiovese fluorescently stained and observed under an optical at 0-, 1-, 3-, and 7-days post inoculum (dpi) of each treatment. From top to bottom: leaves treated with water (U^−^/*Pv*^−^), treated with UPSIDE^®^ (U^+^/*Pv*^−^), inoculated with *Plasmopara viticola* (U^−^/*Pv*^+^), and treated with UPSIDE^®^ and inoculated with *P. viticola* (U^+^/*Pv*^+^). Scale bar = 50 µm.

No fluorescence-stained *Pv* structures were observed in plants treated with sterile water and mock-inoculated (U^−^/*Pv*^−^) or in plants treated with U and mock-inoculated (U^+^/*Pv*^−^; Figure 1). In plants treated with sterile water and inoculated with *Pv* (U^−^/*Pv*^+^), the oomycete structures were detectable at 3 days post inoculum (dpi), and the oomycete was fully sporulated at 7 dpi. In contrast, in plants treated with U and inoculated with *Pv* (U^+^/*Pv*^+^), no downy mildew signs were observed.

### 2.3. Biochemical Results

**Figure 2:**
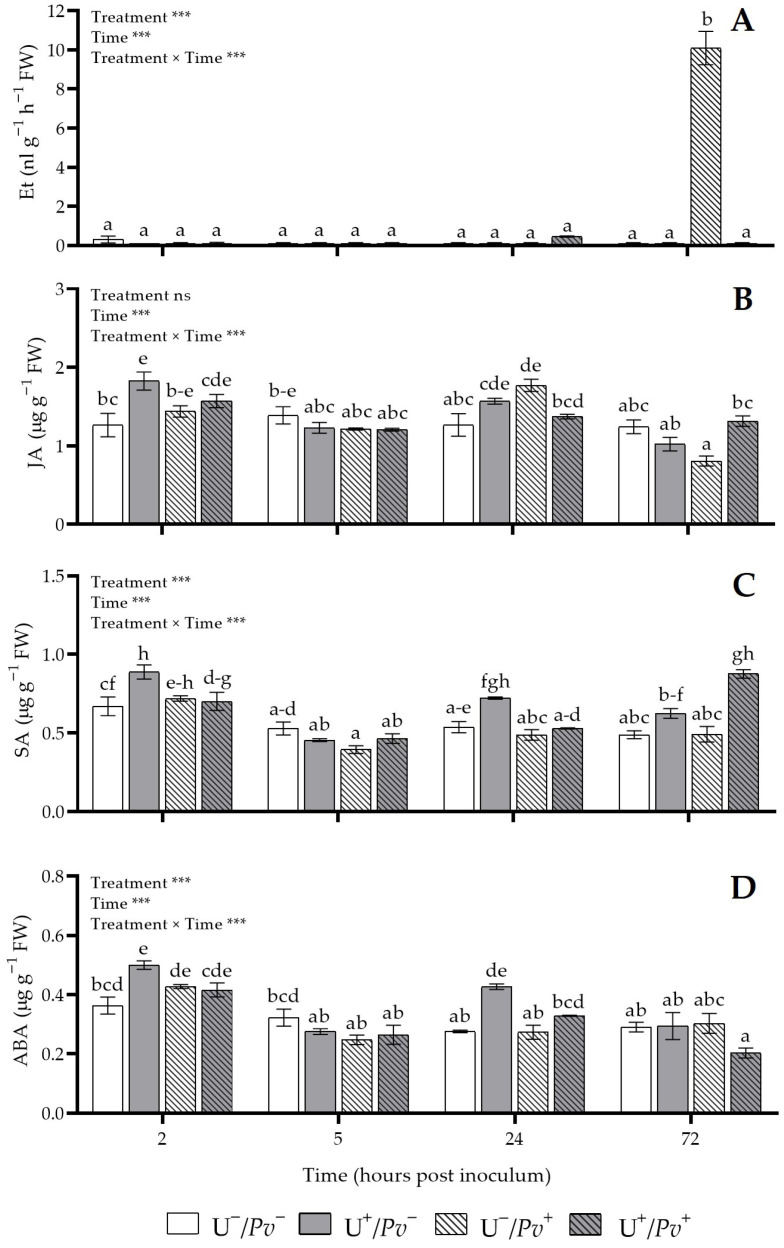
Variation in ethylene (Et; (**A**)), jasmonic acid (JA; (**B**)), salicylic acid (SA; (**C**)) and abscisic acid (ABA; (**D**)) content in *Vitis vinifera* cv. Sangiovese leaves treated with water (U^−^/*Pv*^−^, white bar), treated with UPSIDE^®^ (U^+^/*Pv*^−^, grey bar), inoculated with *Plasmopara viticola* (U^−^/*Pv*^+^, white dashed bar), and treated with UPSIDE^®^ and inoculated with *P. viticola* (U^+^/*Pv*^+^, grey dashed bar). Leaves were harvested at 2-, 5-, 24-, and 72-h post inoculum. At each sampling time, at least five plants were analyzed. Data are presented as mean ± standard error (n = 5). And *p* values for the effects of the factors “treatment” and “time”, and their interaction, obtained from a two-way analysis of variance, are reported (*** *p* ≤ 0.001, ns *p* > 0.05). In each graph, different letters indicate significant differences among means according to Tukey’s HSD post hoc test. Abbreviation: FW, fresh weight.

The effects of “treatment”, “time”, and their interaction were significant for all the phytohormones analyzed [i.e., ethylene, jasmonic acid, salicylic acid, and abscisic acid (Et, JA, SA, and ABA, respectively)], except for the “treatment” factor for JA (Figure 2). A significant peak of Et was detected at 72 h post inoculum (hpi) in U^−^/*Pv*^+^ plants compared with all other plant groups (10.09 ± 0.85 vs. 0.0016 ± 0.0001 nl g^−1^ h^−1^ fresh weight, FW; Figure 2A). No significant changes were observed at the other sampling times. Jasmonic acid levels increased in U^+^/*Pv*^−^ at 2 hpi (+44% compared with U^−^/*Pv*^−^; Figure 2B), and in U^−^/*Pv*^+^ at 24 hpi (+40% compared with U^−^/*Pv*^−^). By contrast, at 72 hpi, JA levels were 39% lower in U^−^/*Pv*^+^ than in U^+^/*Pv*^+^ plants (Figure 2B). No significant differences were detected at the remaining sampling times. Salicylic acid content increased in U^+^/*Pv*^−^ plants at 2 hpi compared with both U^−^/*Pv*^−^ and U^+^/*Pv*^+^ (+33 and +27%, respectively), and at 24 hpi compared with U^−^/*Pv*^+^ and U^+^/*Pv*^+^ (+48 and +37%, respectively; Figure 2C). At 72 hpi, SA reached its highest levels in U^+^/*Pv*^−^ plants compared with all other groups (+66% on average). No significant differences were observed at 5 hpi (Figure 2C). An increase in ABA was observed in U^+^/*Pv*^−^ plants at both 2 (+38% compared with U^−^/*Pv*^−^) and 24 hpi (+47% on average compared with U^−^/*Pv*^−^, U^−^/*Pv*^+^, and U^+^/*Pv*^+^; Figure 2D). No significant changes were recorded at the remaining sampling times.

**Figure 3:**
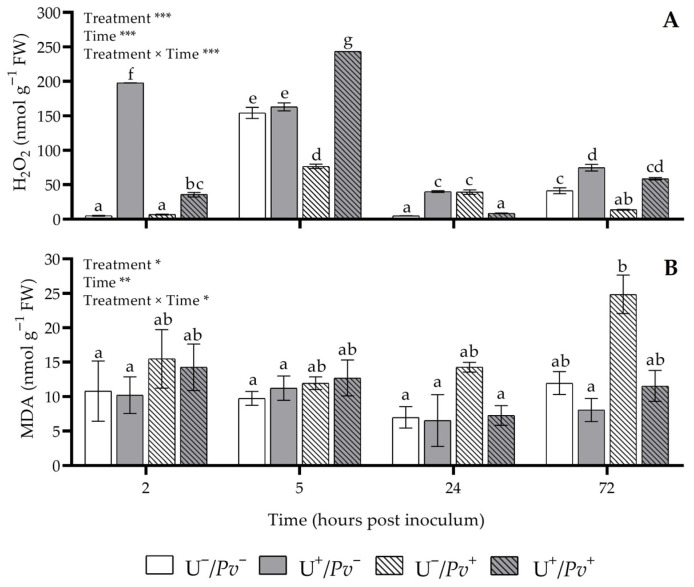
Variation in hydrogen peroxide (H_2_O_2_; (**A**)) and malondialdehyde (MDA; (**B**)) content in *Vitis vinifera* cv. Sangiovese leaves treated with water (U^−^/*Pv*^−^, white bar), treated with UPSIDE^®^ (U^+^/*Pv*^−^, grey bar), inoculated with *Plasmopara viticola* (U^−^/*Pv*^+^, white dashed bar), and treated with UPSIDE^®^ and inoculated with *P. viticola* (U^+^/*Pv*^+^, grey dashed bar). Leaves were harvested at 2-, 5-, 24-, and 72-h post inoculum. At each sampling time, at least five plants were analyzed. Data are presented as mean ± standard error (n = 5). And *p* values for the effects of the factors “treatment” and “time”, and their interaction, obtained from a two-way analysis of variance, are reported (*** *p* ≤ 0.001, ** *p* ≤ 0.01, * *p* ≤ 0.05). In each graph, different letters indicate significant differences among means according to Tukey’s HSD post hoc test. Abbreviation: FW, fresh weight.

The effects of “treatment”, “time”, and their interaction were significant for both hydrogen peroxide (H_2_O_2_) and malondialdehyde (MDA; Figure 3). At 2 hpi, H_2_O_2_ levels markedly increased in both U^+^/*Pv*^−^ (39-, 29-, and 6-fold higher than in U^−^/*Pv*^−^, U^−^/*Pv*^+^, and in U^+^/*Pv*^+^, respectively), and in U^+^/*Pv*^+^ (7- and 5-fold higher than in U^−^/*Pv*^−^ and U^−^/*Pv*^+^; Figure 3A). Similarly, at 5 hpi, H_2_O_2_ levels were significantly higher in U^+^/*Pv*^+^ plants (+58 and +49% compared with U^−^/*Pv*^−^ and U^+^/*Pv*^−^, respectively, and 3-fold higher than in U^−^/*Pv*^+^). In contrast, H_2_O_2_ levels decreased in U^−^/*Pv*^+^ in plants (−57% on average compared with the other plant groups). At 24 hpi, elevated H_2_O_2_ levels were observed in both U^+^/*Pv*^−^ and U^−^/*Pv*^+^ plants, exceeding those measured in U^−^/*Pv*^−^ and U^+^/*Pv*^+^ by more than 20- and 4-fold on average, respectively. At 72 hpi, H_2_O_2_ levels increased again in U^+^/*Pv*^−^ plants (+82% and more than 5-fold compared with U^−^/*Pv*^−^ and U^−^/*Pv*^+^, respectively), whereas a marked decrease was recorded in U^−^/*Pv*^+^ plants (−75% on average compared with the other plant groups). Regarding MDA, no significant changes were detected throughout the experiment, except at 72 hpi, when a more than 2-fold increase was observed in U^−^/*Pv*^+^ plants compared with U^+^/*Pv*^−^ (Figure 3B).

### 2.4. Molecular Results

**Figure 4:**
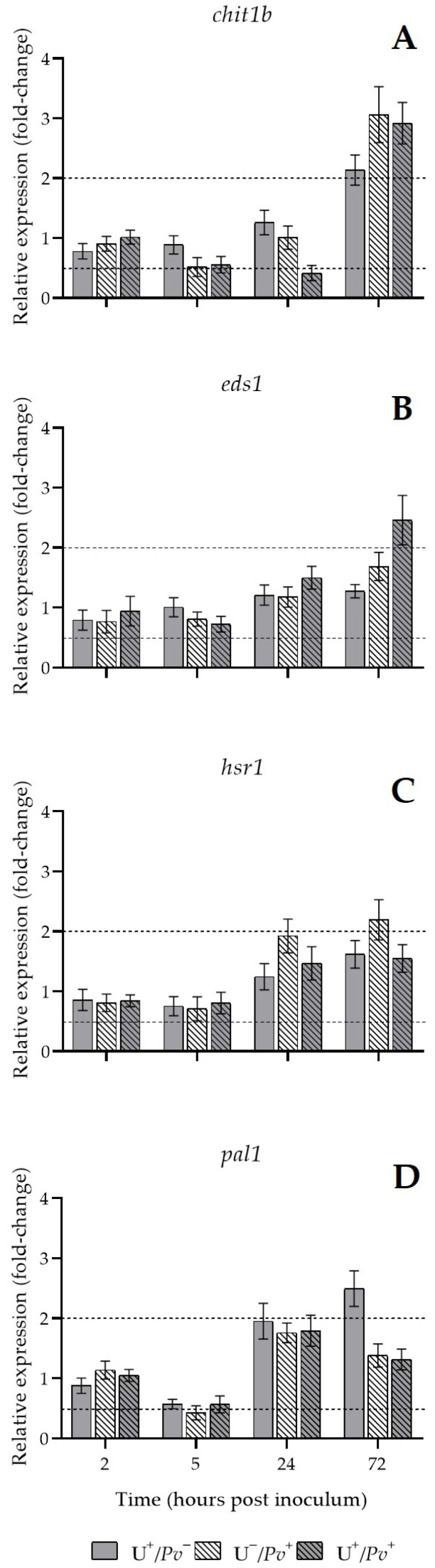
Relative expression of chitinase 1b (*chit1b*; (**A**)), enhanced disease susceptibility 1 (*eds1*; (**B**)), hypersensitive response marker 1 (*hsr1*; (**C**)), and phenylalanine-ammonia lyase (*pal1*; (**D**)) encoding genes of *Vitis vinifera* cv. Sangiovese leaf treated with UPSIDE^®^ (U^+^/*Pv*^−^, grey), inoculated with *Plasmopara viticola* (U^−^/*Pv*^+^, white dashed), and pre-treated with UPSIDE^®^ and inoculated with *P. viticola* (U^+^/*Pv*^+^, grey dashed) at 2-, 5-, 24-, and 72-h post inoculum in comparison with untreated leaves (basal condition 2^−ΔΔCt^ = 1).

Application of U and/or inoculation with *Pv* induced changes in gene expression in U^+^ and/or *Pv*^+^ leaves compared with U^−^/*Pv*^−^ plants. At 24 hpi, chitinase 1 b (*chit1b*) was downregulated in U^+^/*Pv*^+^ plants (0.42-fold). However, at 72 hpi, *chit1b* was upregulated in U^+^/*Pv*^−^, U^−^/*Pv*^+^, and U^+^/*Pv*^+^ plants (more than 2-, 3-, and 3-fold, respectively, compared with U^−^/*Pv*^−^; Figure 4A). Similarly, enhanced disease susceptibility 1 (*eds1*) was overexpressed in U^+^/*Pv*^+^ plants at 72 hpi (3-fold compared with U^−^/*Pv*^−^; Figure 4B). In contrast, hypersensitive response 1 (*hsr1*) was upregulated in U^−^/*Pv*^+^ plants at 72 hpi (2-fold compared with U^−^/*Pv*^−^; Figure 4C). Phenylalanine-ammonia lyase (*pal1*) was downregulated in U^−^/*Pv*^+^ plants at 5 hpi (2-fold relative to U^−^/*Pv*^−^), whereas it was upregulated in U^+^/*Pv*^−^ plants at 72 hpi (3-fold compared with U^−^/*Pv*^−^; Figure 4D).

**Figure 5:**
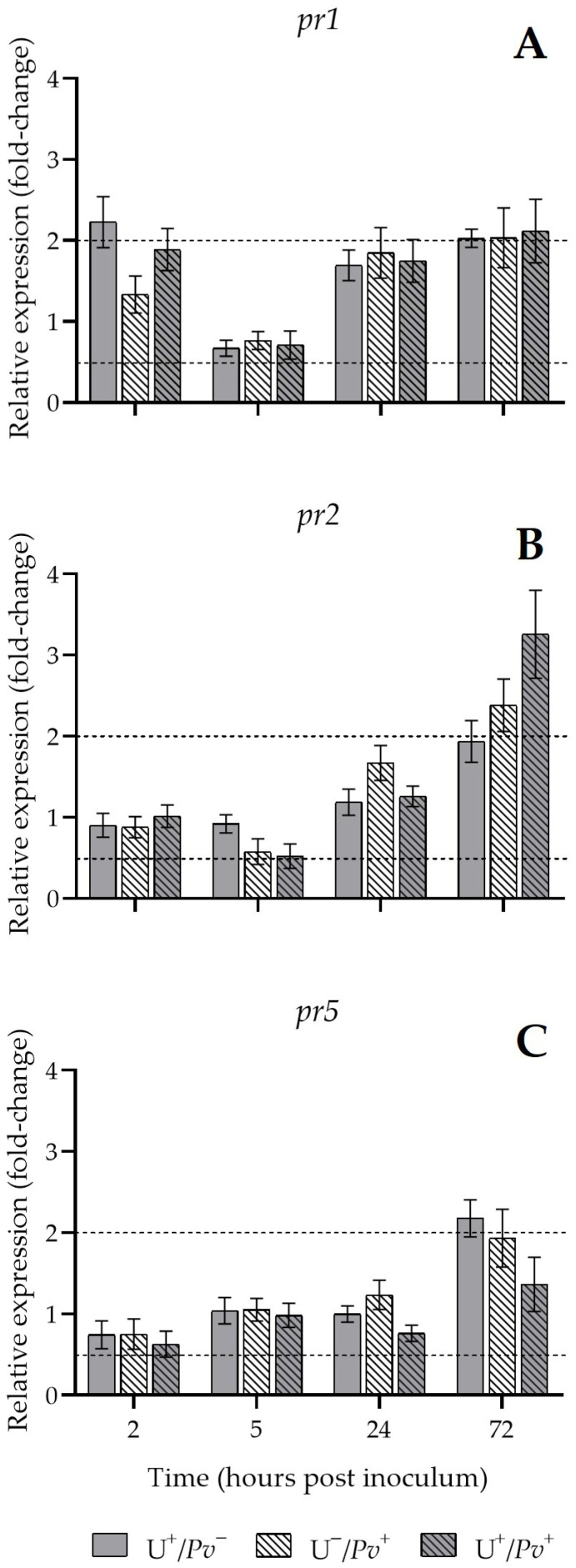
Relative expression of pathogenesis related 1 (*pr1*; (**A**)), pathogenesis related 2 (*pr2*; (**B**)), and pathogenesis related 5 (*pr5*; (**C**)) encoding genes of *Vitis vinifera* cv. Sangiovese leaf treated with UPSIDE^®^ (U^+^/*Pv*^−^, grey), inoculated with *Plasmopara viticola* (U^−^/*Pv*^+^, grey dashed), and pre-treated with UPSIDE^®^ and inoculated with *P. viticola* (U^+^/*Pv*^+^, white dashed) at 2-, 5-, 24-, and 72-h post inoculum in comparison with untreated leaves (basal condition 2^−ΔΔCt^ = 1).

An upregulation of *pr1* was observed at 2 hpi in U^+^/*Pv*^−^ plants (2-fold) and at 72 hpi in all plant groups (approximately 2-fold compared with U^−^/*Pv*^−^; Figure 5A). Similarly, a marked increase in *pr2* expression was detected in U^−^/*Pv*^+^ and U^+^/*Pv*^+^ plants (2- and 3-fold; Figure 5B), whereas *pr5* was upregulated only in U^+^/*Pv*^−^ plants (2-fold compared with U^−^/*Pv*^−^; Figure 5C).

## 3. Discussion

Microbial extract-based plant resistance inducers have been shown to activate crosstalk between SAR and ISR. Among these compounds, cerevisane, consisting of cell wall components from a selected *S. cerevisiae* strain, has been proposed as a resistance inducer in grapevine and other horticultural crops against fungal pathogens [25]. In this context, elicitor-based approaches are increasingly promising tools for sustainable viticulture, as they may complement or partially replace conventional fungicide-based disease control strategies. As a yeast-derived product, cerevisane likely contains multiple MAMPs and/or elicitors capable of triggering complex defense signaling cascades when applied to leaf surfaces [18,26]. Indeed, phytohormones such as Et, SA, and ABA, and the signaling molecule JA, all of which play key roles in plant defense regulation, have been reported to be activated by cerevisane [27]. While Et and JA are typically associated with resistance to necrotrophic pathogens (although they also contribute to defense against *Pv*), SA is primarily involved in responses to biotrophic pathogens.

In this study, we evaluated the effectiveness of a novel *S. cerevisiae*-based product (U) as an enhancer of grapevine defense responses against the causal agent of downy mildew. Application of U induced an early accumulation of JA, SA, and ABA at 2 hpi, suggesting that these phytohormones/signaling molecules are involved in U-mediated priming responses. This early hormonal activation was followed by increased SA and ABA levels at 24 hpi, which were associated with H_2_O_2_ production (detected at 2, 24, and 72 hpi), potentially establishing a feedback loop [28]. The observed increase in H_2_O_2_ likely reflects an early oxidative burst that acts as a key signaling event, activating downstream defense pathways and contributing to the priming of plant immune responses. These hormonal dynamics suggest a coordinated defense response in which early JA and ABA signaling may contribute to priming and signaling crosstalk, while SA appears to play a central role in the establishment of effective resistance at later stages (as supported by its association with downstream defense gene activation and disease suppression). In this context, H_2_O_2_ likely acts a key signaling hub, modulating the crosstalk between SA- and JA-dependent pathways and contributing to the coordination of defense responses leading to enhanced resistance, including hypersensitive response (HR) and resistance establishment. Notably, no enhancement of Et was observed throughout the experimental period, indicating that this hormone was not directly involved in U-mediated priming responses [29]. The sustained accumulation of H_2_O_2_ (except at 5 hpi) suggests that U application did not directly generate H_2_O_2_ but rather triggered stress-related H_2_O_2_ formation [30]. A distinct temporal pattern of phytohormone activation was observed in U^−^/*Pv^+^* plants. In this case, H_2_O_2_ accumulation at 24 hpi preceded Et synthesis at 72 hpi and was accompanied by a marked increase of JA at 24 hpi. These results confirm a crosstalk between JA- and Et-dependent signaling pathways during lesion spread and propagation to surrounding cells after *Pv* infection. Elevated H_2_O_2_ levels may contribute to HR activation but could also influence *Pv* development through modulation of redox status, as confirmed by the reduction of H_2_O_2_ content at 5 and 72 dpi [20]. The absence of SA enhancement throughout the experiment indicates that SA was not centrally involved in programmed cell death or associated signaling pathways in this interaction [31]. In plants subjected to sequential double-treatments (U^+^/*Pv*^+^), a specific hormonal crosstalk was observed. Early H_2_O_2_ induction (2 and 5 hpi) was followed by SA accumulation at 72 hpi, indicating that SA participated in regulating defense responses and HR activation. This was consistent with the observed reduction in disease severity and the absence of *Pv* structures on leaf surfaces at 7 dpi. These results suggest that the early hormonal reprogramming (2–24 hpi) contributes to priming the plant defense system, thereby enabling a more efficient response to *Pv* infection and ultimately leading to reduced disease severity at later stages. This is consistent with the reduced presence of *Pv* structures observed on leaf surfaces, indicating a clear link between early hormonal responses and the limitation of pathogen development. Overall, U-induced priming appears to rely on early signaling events, including phytohormone/signaling molecules and ROS accumulation, which enhance the speed, amplitude, and effectiveness of SA-mediated defense responses upon *Pv* infection. In this context, SA appears to function as a mediator of defense-related metabolic pathways, such as the phenylpropanoid pathway, rather than solely as a signaling molecule [32]. The divergence in hormonal profiles and peak magnitudes among single and double treatments highlights the complexity of the transcriptional regulatory network activated by U priming. Such host-mediated activation of defense responses may contribute to reducing dependence on synthetic fungicide application in integrated disease management strategies.

At the genic level, priming alters transcript and protein abundances and/or functionality, involving hormone-responsive marker genes and/or defense-related pathways [33]. Many SAR-related genes participate in defense activation through either rapid local recognition of pathogen-derived molecules or longer-lasting systemic responses. In U^+^/*Pv*^−^ plants, *pr1* expression was rapidly induced at 2 hpi, indicating involvement in early defense activation. Sustained overexpression of *pr1*, *chit1b*, and *pal1* at 72 hpi confirms that U activates multiple defense pathways, including those overlapping with HR and SAR. The transient downregulation of *pal1* at 5 hpi (encoding a key enzyme of the phenylpropanoid pathway) may reflect its participation in an early, quick, and local defense response [34]. In U^−^/*Pv^+^* plants, genes associated with pathogen recognition (e.g., *pr1* and *pr2*) and defense-related pathways (e.g., *chi1b* and *hrs1*) were upregulated at 72 hpi, supporting their role in systemic defense activation. This shift from early local responses (as indicated by *pal1* downregulation at 5 hpi) toward systemic activation may reflect progressive pathogen colonization. HR-related genes are typically activated following an oxidative burst, consistent with the observed ROS dynamics [29]. In U^+^/*Pv^+^* plants, SA-responsive genes (e.g., *pr1*, *pr2*, and *eds1*) and defense-related genes (*chit1b*) were overexpressed at 72 hpi. Pathogenesis-related (PR) proteins function to limit pathogen multiplication and spread, and their induction is generally mediated by low SA concentrations [35], consistent with the delayed SA accumulation observed in this study. Although PR induction is considered a relatively late HR event, it contributes significantly to resistance establishment [36]. Moreover, *eds1* upregulation aligns with increased SA levels, as this gene is a key regulator of SA-dependent defense signaling in grapevine [37]. Despite the comprehensive multi-level approach adopted in this study, some limitations should be acknowledged. First, the experiments were carried out under controlled conditions, which may not fully reflect the complexity and variability of the open field, where multiple biotic and abiotic stress factors can influence plant responses. Second, the study focused on a selected set of phytohormones/signaling molecules, oxidative stress markers, and defense-related genes, and therefore does not capture the full extent of the regulatory networks involved in U-mediated priming. In addition, although the observed functional and gene-level changes are consistent with enhanced resistance, the causal relationship between these responses and disease suppression remains to be fully elucidated. Compared to conventional fungicides, U acts as a plant resistance inducer rather than a direct antimicrobial agent. This mode of action may support more sustainable disease management strategies by reducing reliance on synthetic fungicides and potentially lowering environmental impact and selection pressure on pathogen populations. UPSIDE^®^ has no impact on any beneficial organisms and has no residues, being a great alternative product to copper (also in relation to the diverse modes of action in the spray programs). It is worth noting that biologicals often struggle with low efficacy; therefore, it is crucial to study modes of action to better understand the product and thus be able to fully explore its potential.

## 4. Conclusions

Our findings indicate that U application acts as preventive priming stimulus, enabling grapevine plants to mount a more efficient defense response upon pathogen challenge. In the absence of U, *Pv* infection triggered JA-Et driven signaling and upregulation of *pr1*, *pr2*, *chit1b*, and *hsr1*; however, this response was insufficient to prevent disease development. In contrast, plants subjected to U training and inoculated with *Pv* exhibited a distinct and enhanced SA-centered response, accompanied by upregulation of *pr1*, *pr2*, *chit1b*, and *eds1*, ultimately leading to effective disease suppression and the absence of downy mildew structures. Additionally, the leaf disc assay indicated that U may exert a direct inhibitory effect on *Pv* development; however, further experiments are required to clearly establish the potential direct effect of the product—as the assays conducted so far provided only preliminary evidence. Future studies should investigate the molecular mechanisms underlying U-induced priming, including the identification of active elicitor components, their perception by plant receptors, and the downstream signaling pathways involved. Integrative omics approaches (e.g., transcriptomics, proteomics, and metabolomics) would be particularly valuable for elucidating the regulatory networks associated with priming. Moreover, validation under field conditions and across different grapevine cultivars will be essential to confirm the practical applicability of the treatment. Its successful integration into commercial grapevine management will also depend on optimization of application strategies, compatibility with existing plant protection programs, and its economic and practical feasibility. In this context, priority should be given to long-term field studies aimed at evaluating the persistence and consistency of induced resistance, the effects of repeated applications on plant physiology, productivity, and grape quality, as well as interactions with environmental conditions and vineyard management practices. From a practical perspective, U is expected to be most effective when applied preventively, particularly under environmental conditions favorable to pathogen development. For this reason, Kwizda Agro recommends starting the application at the stage BBCH15 before the onset of disease and then perform two additional applications in the interval 7–10 days until flowering (as this is the most susceptible stage of the plant). Its use within integrated disease management strategies, in combination with conventional fungicides, may improve control efficacy while contributing to a reduction in chemical inputs. Taken together, these results support the potential of U as a promising and environmentally sustainable tool for integration into both conventional and organic viticulture.

## 5. Materials and Methods

### 5.1. Leaf Disc Assay

An in vitro test was conducted to evaluate the potential of U to inhibit *Pv* development, following the method described by Scimone et al. [38], with modifications according to [39]. It is worth nothing that U is a standardized *S. cerevisiae*-derived elicitor preparation provided by Kwizda Agro. It is a registered plant protection product intended to be used as fungicide to control *Pv* in vines. It is a suspension concentrate formulation containing active substance ABE-IT 56 (lysate of *S. cerevisiae* strain DDSF623) in concentrations of 325.6 g L^−1^ (29.6 *w*/*w*). Ten leaves were collected from disease-free grapevines, washed under tap water to remove surface debris, and allowed to air-dry. Half of the leaves were sprayed on both surfaces until runoff with U diluted in sterile H_2_O at the concentration recommended by the manufacturer (2.5 mL L^−1^ *v*/*v*), which has been shown to be non-phytotoxic [18,22], using a hand sprayer, while the remaining leaves were sprayed with sterile water (control set). Leaf discs were excised by using a cork borer (∅ 18 mm), obtaining 16 discs per treatment. Discs were placed abaxial side up on Petri dishes containing water agar (6 g L^−1^, *w*/*v*; Sigma, Milan, Italy), with four leaf discs per plate. Each leaf disc was inoculated with *Pv* using a hand sprayer. The inoculum suspension was prepared from downy mildew-infected grapevine leaves collected in the field and rinsed with sterile water. Sporangial concentration was determined using a hemocytometer (Henneberg-Sander, Giessen Lützellinden, Germany) and adjusted to 10^5^ sporangia mL^−1^. After inoculation, leaf discs were incubated at room temperature for 10 days. The percentage of each disc covered in sporulating downy mildew was assessed under a stereomicroscope (Leica S9i, Leica, Wetzlar, Germany).

### 5.2. Plant Material and Experimental Design

In July 2023, 48 five-year-old plants of *V. vinifera* cv Sangiovese (clone F9-A5-48; rootstock 110 Richter; purchased from a local nursery) were maintained in a greenhouse facility owned by the Department of Agriculture, Food, and Environment of the University of Pisa, at San Piero a Grado (Pisa, Italy). After a two-week acclimation period, plants were divided into two groups. Twenty-four grapevines were foliar-sprayed with U (U^+^; 2.5 mL L^−1^, *v/v* in water) once a week for three consecutive weeks [18], while the remaining 24 plants were sprayed with sterile water and used as control (U^−^). At the end of the treatment period, twelve plants per group were uniformly spray-inoculated with a *Pv* sporangial suspension prepared as described above. Sterile water was used for mock inoculation (*Pv*^−^). The four experimental groups were therefore designated as follows: U^−^/*Pv*^−^, U^+^/*Pv*^−^, U^−^/*Pv*^+^, and U^+^/*Pv*^+^, respectively. Untreated and water-treated plants were included as controls to establish baseline biochemical and molecular responses. These controls allowed discrimination of the effects of U application and/or *Pv* inoculation from natural variability and background plant responses. Fully expanded leaves were harvested from five plants per group at 2, 5, 24, and 72 hpi and immediately used for Et determination. Sampling times were selected based on the infection dynamics of *Pv* in grapevine leaves, encompassing early post-penetration stages (2–5 hpi), pathogen establishment with substomatal vesicle formation (24 hpi), and subsequent active colonization (72 hpi) [1,40]. Leaf material was ground in liquid nitrogen and stored at −80 °C until further biochemical and molecular analyses. For microscopic observations aimed at monitoring disease development, separate sampling times were adopted and leaves were collected at 0, 1, 3, and 7 dpi.

### 5.3. Microscopic Observation

*Plasmopara viticola* structures developed on the leaf surface of *V. vinifera* were fluorescently stained according to [41] to investigate the infection process after inoculation. Leaf tissue segments (approximately 1 cm in length) excised from sampled leaves were immersed in a solution containing 70% ethanol (*v*/*v*, in water) and fluorescent brightener 28 (Sigma, Milan, Italy). After 1 h of incubation, samples were fixed on glass slides in 50% glycerol and visualized under a fluorescence optical microscope (DM 4000^®^ B led, Leica, Wetzlar, Germany). Photomicrographs were captured using a Canon PowerShot S50^®^ camera (Canon Italia, Milan, Italy).

### 5.4. Biochemical Analysis

Ethylene emission was determined following [18]. Fresh whole leaves were enclosed in 15 mL glass vials and incubated for 2 h. Subsequently, 1 mL of headspace was withdrawn using a hypodermic syringe and injected into an Agilent 8890B gas chromatograph (GC) equipped with an Agilent HP-PLOT/Q+PT capillary column (30 m × 0.32 mm; coating thickness 20 μm) and an Agilent 5977B single quadrupole mass detector (MS; Agilent Technologies Inc., Santa Clara, CA, USA). Helium was used as carrier gas at a flow rate of 1 mL min^−1^. The injector and the transfer line were set at 180 °C. The oven temperature was maintained at 36 °C. The ion source and quadrupole temperatures were set at 230 and 150 °C, respectively. Mass spectra were acquired in electron impact mode at 70 eV. Ethylene quantification was performed in selected-ion monitoring mode at *m/z* 28 using MassHunter Workstation (version 10.0, Agilent Technologies Inc.).

Jasmonic acid, SA, and ABA were determined according to [42], with minor modifications. Frozen leaf samples (200 mg) were extracted with 1.5 mL of 80% methanol (MeOH; *v/v* in H_2_O). After 2 h of incubation on ice, samples were sonicated and centrifuged (15,000× *g* at 4 °C for 20 min). Supernatants were filtered and evaporated at 35 °C under vacuum (RVC 2-25 CDplus, Martin Christ Gefriertrocknungsanlagen GmbH, Germany). Residues were re-suspended in 40 μL of ethyl acetate and injected into the previously described GC-MS system equipped with an Agilent DB-1MS (UI) capillary column (30 m × 0.25 mm; coating thickness 0.25 μm). Helium was used as carrier gas at 1 mL min^−1^. The injector and the transfer line were set at 250 °C. The temperature program was as follows: the initial column temperature was set at 90 °C for 2 min, increasing to 300 °C at 10 °C min^−1^. Source and quadrupole temperatures were set at 230 and 150 °C, respectively. The mass data were collected in the electron impact mode at 70 eV with a scan range of 40–500 *m/z*, and the plant hormone quantification was performed at the selected-ion monitoring mode at *m/z* 210 (JA), 120 (SA), and 190 (ABA) amu by using the MassHunter Workstation.

Hydrogen peroxide content was measured using the Amplex^TM^ Red Hydrogen Peroxide/Peroxidase Assay Kit (Molecular Probes, Life Technologies Corp., Carlsbad, CA, USA), according to [43]. Frozen samples (50 mg) were extracted in 1 mL of 20 mM potassium-phosphate buffer (pH 6.5) and incubated for 30 min at 25 °C in the dark. Fluorescence was measured using a Victor3–1420 Multilabel Couter microplate reader (Perkin Elmer Inc., Waltham, MA, USA), using 530 nm excitation and 590 nm emission wavelengths for resorufin. Concentrations were calculated from a standard curve ranging from 0 to 20 μM H_2_O_2_.

Oxidative damage was quantified by measuring lipid peroxidation using the thiobarbituric acid-reactive substances (TBARS) assay, based on MDA formation, following [44], with minor modifications. Frozen samples (100 mg) were homogenized in 1 mL of 0.1% trichloroacetic acid (TCA; *w*/*v*, in water). A 200 µL aliquot of the homogenate was mixed with (i) 300 µL of 0.1% TCA (*w*/*v*, in water) and 500 mL of 20% TCA (*w*/*v*, in water); (ii) 300 µL of 0.1% TCA (*w*/*v*, in water) and 500 µL of 0.5% thiobarbituric acid (*w*/*v*, in 20% TCA). Samples were incubated for 30 min at 95 °C, then centrifuged at 10,000× *g* for 10 min. Absorbance was measured at 532 nm using the same microplate reader described above, with corrections for nonspecific turbidity by subtracting readings at 440 and 600 nm.

To ensure consistency across different time points, all samples were collected and processed under identical experimental conditions, including extraction protocols and analytical settings. Analyses were performed on biological replicates for each treatment and time point, and quantification was based on standard calibration curves to ensure accuracy, reproducibility, and comparability of the data.

### 5.5. Molecular Analysis

The relative expression of the plant defense-related genes was evaluated by quantitative Real-Time PCR by using a Rotor-Gene Q cycler (Qiagen, Milan, Italy). Seven genes were selected to investigate the resistance mechanisms activated in the experimental plant groups: one gene involved in basal resistance, i.e., phenylalanine-ammonia lyase (*pal1*); four genes associated with the SA-depending signaling, i.e., pathogenesis-related proteins 1, 2, and 5 (*pr1*, *pr2,* and *pr5*), and enhanced disease susceptibility 1 (*eds1*); two genes involved in JA/Et-depending signaling, i.e., hypersensitive response marker 1 (*hsr1*), chitinase encoding gene (*chit1b*). Their relative expression was evaluated by quantitative Real-Time PCR by using the Rotor-Gene Q cycler (Qiagen, Milan, Italy) as reported in [18], with minor modifications. Specifically, RNA was extracted from leaf material by using the RNeasy Mini Kit (Qiagen, Milan, Italy). A total of 400 ng of DNase I-treated RNA was used to synthesize cDNA with the Maxima First Strand cDNA Synthesis Kit (K1642, Applied Biosystems, Monza, Italy) for RT-qPCR. All PCR reactions were set up with cDNA, QuantiNova SYBR^®^ Green PCR Master Mix 2X (Qiagen, Milan, Italy), each primer (details are reported in [18]) and Nuclease-Free water. Amplifications were carried out under the following cycling conditions: initial activation at 95 °C for 2 min; 40 cycles of denaturation at 95 °C for 5 s, and combined annealing/extension at 60 °C for 10 s. Cycle threshold (CT) values were determined with Rotor-Gene Q Series Software v2.3.1 (Qiagen, Milan, Italy), using the pyruvate-decarboxylase 1 (*pcd1*) gene as endogenous control, as its expression remained stable across the experimental treatments [18], likely due to its central role in primary carbon metabolism [43]. Gene expression levels were calculated as 2^−ΔΔCt^ [45]. Primers were designed with the Geneious 10.0.9 (www.geneious.com, accessed on 1 September 2021), and in silico analyzed using the NetPrimer tool (www.premierbiosoft.com/NetPrimer/AnalyzePrimer.jsp; accessed in 30 September 2021). Primers utilized for gene expression analysis were evaluated for amplification efficiency and potential dimer formation, with details provided in Table S1.

To ensure consistency among treatments, all samples were processed under identical experimental conditions, including RNA extraction, cDNA synthesis, and RT-qPCR setup. Comparisons among treatments were conducted within each sampling time, ensuring accuracy, reproducibility, and comparability of the results.

### 5.6. Statistical Analysis

The Shapiro–Wilk’s and Levene’s tests were used to assess the normal distribution of data and the homogeneity of variance. The results from biochemical analyses were submitted to a two-way ANOVA to evaluate the effects of “treatment”, “time”, and their interaction. When significant interactions were detected, Tukey’s HSD post hoc comparisons were used to identify differences among treatments and time points, enabling a comprehensive assessment of early responses associated with priming effects. The results from molecular analyses were submitted to a one-way ANOVA performed separately for each sampling time point to evaluate the effect of “treatment”. When significant differences were detected, Tukey’s HSD post hoc test was applied to compare means among treatments within each time point, allowing the identification of temporal patterns in gene expression associated with U-induced priming. Comparisons among means were determined by using JMP Pro 14.0 software (SAS Institute Inc., Cary, NC, USA). For all the analyses, statistically significant effects were considered for *p* ≤ 0.05.

## Data Availability

The raw data supporting the conclusions of this article will be made available by the authors without undue reservation.

## Supplementary Materials

The following supporting information can be downloaded at https://www.mdpi.com/article/10.3390/plants15091297/s1. Figure S1. Visual assessment of *Vitis vinifera* cv Sangiovese leaf disks sprayed with water (U^−^) or UPSIDE (U^+^), and inoculated with *Plasmopara viticola* (*Pv*^+^). Table S1. Primer sequences used for gene expression analysis of *Vitis vinifera* cv. Sangiovese defence-related genes (5′ to 3′).

## Abbreviations

The following abbreviations are used in this manuscript:

ABA	Abscisic acid
ANOVA	Analysis of variance
*chit1b*	Chitinase 1b
CT	Cycle threshold
DAMPs	Damage-associated molecular patterns
dpi	days post inoculum
*eds1*	Enhanced disease susceptibility 1
Et	Ethylene
GC	Gas chromatograph
H_2_O_2_	Hydrogen peroxide
hpi	Hours post inoculum
HR	Hypersensitive response
*hsr1*	Hypersensitive response marker 1
ISR	Induced systemic resistance
JA	Jasmonic acid
MAMPs	Microbe-associated molecular patterns
MDA	Malondialdehyde
MeOH	Methanol
MS	mass spectrometer
*pal1*	Phenylalanine-ammonia lyase
*pr1*	Pathogenesis-related protein 1
*pr2*	Pathogenesis-related protein 2
*pr5*	Pathogenesis-related protein 5
*Pv*	*Plasmopara viticola*
SA	Salicylic acid
SAR	Systemic acquired resistance
TBARS	Thiobarbituric acid-reactive-substances
TCA	Trichloroacetic acid
U	UPSIDE^®^
