# Supplementary material for: Harnessing the Action Model of the Defense Responses Induced by UPSIDE® Against Plasmopara viticola in Grapevine
Source: Plants (Basel). 2026 Apr 23;15(9):1297. doi: 10.3390/plants15091297 (PMC13164691; doi:10.3390/plants15091297)
Supplement: Supplementary file 1 [file plants-15-01297-s001.zip › plants-4259986-supplementary.pdf]

Supplementary material

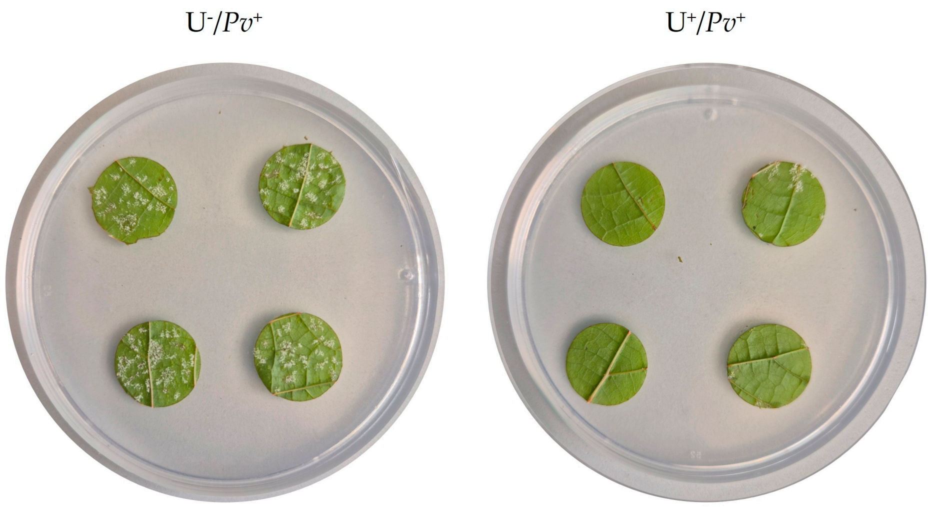

Figure S1. Visual assessment of *Vitis vinifera* cv Sangiovese leaf disks sprayed with water (U$^-$) or UPSIDE (U$^+$), and inoculated with *Plasmopara viticola* (*Pv*$^+$).

Table S1. Primer sequences used for gene expression analysis of *Vitis vinifera* cv. Sangiovese defence-related genes (5′ to 3′).

Gene	Name	Sequence	Amplicon (bp)	Efficiency
Piruvate-decarboxylase 1 (*pdc1*)	VPCDFw4	CAGGACCCATTTTCAACGAT	105	1.09
	VPCDRv4	CCATTACCAATCACAACCCG		
Phenylalanine ammonia-lyase 1 (*pal1*)	VPALFw2	GCCAATCCTGTCACCAACCA	173	0.98
	VPALRv2	CCTCCAAATGCCTCAAATCAA		
Chitinase 1B (*chit1b*)	VCHITBFw2	TCCAGCAGGAAAAGCCATAAACTA	104	0.93
	VCHITBRv2	GGGTCATCCAGAACCACAAAGC		
Hypersensitive Responsive marker 1 (*hsr1*)	VHSR1Fw2	GCCCCTGCCTCCAATGATT	165	1.06
	VHSR1Rv2	GCCTAAACAGAAACCGCCACC		
Enhaced Disease Susceptibility 1 (*eds1*)	VEDS1Fw1	TGGCTTTGCTATTTTTGCGTT	146	1.01
	VEDS1Rv1	AGGCGTTGACCACAGCGACT		
Pathogenesis Related protein 2 (*pr2*)	VPR2Fw3	GGGACATTCGCCTAGATTATGCT	132	1.05
	VPR2Rv3	CGCCACCAACCCTCTCAAGT		
Pathogenesis Related protein 5 (*pr5*)	VPR5Fw1	CGCACTTAACCAATTCAGCAACTT	141	0.96
	VPR5Rv1	GGGCACTCTCCCACGATGTC		
Pathogenesis Related protein 1 (*pr1*)	VPR1Fw5	GCTGCCTACGCCCAGAACTATG	145	0.99
	VPR1Rv5	GCTTCTCCCCCACCCACAA		
